# 

**Published:** 1906-07-21

**Authors:** 


					0 jKi =
THE HOSPITAL. HLJIf Ur^*\ 21st 1906.
Vol. XL. [a Newspaper!] SATURDAY,
July 21st, 1906.
[ BeeS ] PRICE THREEPENCE

				

